# Treatment of Barth Syndrome by Cardiolipin Manipulation (CARDIOMAN) With Bezafibrate: Protocol for a Randomized Placebo-Controlled Pilot Trial Conducted in the Nationally Commissioned Barth Syndrome Service

**DOI:** 10.2196/22533

**Published:** 2021-05-31

**Authors:** Lucy Dabner, Guido E Pieles, Colin G Steward, Julian P Hamilton-Shield, Andrew R Ness, Chris A Rogers, Chiara Bucciarelli-Ducci, Rosemary Greenwood, Lucy Ellis, Karen Sheehan, Barnaby C Reeves

**Affiliations:** 1 Bristol Trials Centre (Clinical Trials and Evaluation Unit) Bristol Medical School University of Bristol Bristol United Kingdom; 2 Bristol Congenital Heart Centre University Hospitals Bristol and Weston NHS Foundation Trust Bristol United Kingdom; 3 National Institute of Health Research (NIHR) Biomedical Research Centre (Cardiovascular theme) University Hospitals Bristol and Weston NHS Foundation Trust and University of Bristol Bristol United Kingdom; 4 School of Cellular and Molecular Medicine University of Bristol Bristol United Kingdom; 5 NIHR Biomedical Research Centre (Nutrition theme) University Hospitals Bristol and Weston NHS Foundation Trust and University of Bristol Bristol United Kingdom; 6 Bristol Dental School University of Bristol Bristol United Kingdom; 7 Bristol Heart Institute University Hospitals Bristol and Weston NHS Foundation Trust Bristol United Kingdom; 8 Research Design Service South West National Institute for Health Research Bristol United Kingdom; 9 University Hospitals Bristol and Weston NHS Foundation Trust Bristol United Kingdom

**Keywords:** randomized controlled trial, Barth syndrome, cardiomyopathies, inherited cardiomyopathy, bezafibrate, placebo controlled, rare disease, resveratrol, cardiomyopathy, metabolism, lipid, genetic diseases, x-linked, genes, mitochondrial, controlled clinical trial, placebos, mitochondrial diseases, metabolic diseases, lipid metabolism, lipid metabolism disorders, cross-over studies

## Abstract

**Background:**

Barth syndrome is a rare, life-threatening, X-linked recessive genetic disease that predominantly affects young males and is caused by abnormal mitochondrial lipid metabolism. Currently, there is no definitive treatment for Barth syndrome other than interventions to ameliorate acute symptoms, such as heart failure, cardiac arrhythmias, neutropenia, and severe muscle fatigue. Previous mechanistic studies have identified the lipid-lowering drug bezafibrate as a promising potential treatment; however, to date, no human trials have been performed in this population.

**Objective:**

The aim of this study is to determine whether bezafibrate (and resveratrol in vitro) will increase mitochondrial biogenesis and potentially modify the cellular ratio of monolysocardiolipin (MLCL) to tetralinoleoyl-cardiolipin (L4-CL), ameliorating the disease phenotype in those living with the disease.

**Methods:**

The CARDIOMAN (Cardiolipin Manipulation) study is a UK single-center, double-blinded, randomized, placebo-controlled crossover study investigating the efficacy of bezafibrate in participants with Barth syndrome. Treatment was administered in two 15-week phases with a minimum washout period of 1 month between the phases where no treatment was administered. The primary outcome is peak oxygen consumption (VO_2_ peak). Secondary outcomes include MLCL/L4-CL ratio and CL profile in blood cells, amino acid expression, phosphocreatine to adenosine triphosphate ratio in cardiac muscle and skeletal muscle oxidative function on phosphorus-31 magnetic resonance spectroscopy, quality of life using the Pediatric Quality of Life Inventory questionnaire, absolute neutrophil count, cardiac function and rhythm profiles at rest and during exercise, and mitochondrial organization and function assessments. Outcomes were assessed at baseline and during the final week of each treatment phase.

**Results:**

A total of 12 patients were scheduled to participate across three consecutive research clinics between March and April 2019. In total, 11 participants were recruited, and the follow-up was completed in January 2020. Data analysis is ongoing, with publication expected in 2021.

**Conclusions:**

This trial was approved by the United Kingdom National Research Ethics Service Committee and the Medicines and Healthcare products Regulatory Agency. The feasibility of the CARDIOMAN study will help to inform the future conduct of randomized controlled trials in rare disease populations as well as testing the efficacy of bezafibrate as a potential treatment for the disease and advancing the mechanistic understanding of Barth syndrome.

**Trial Registration:**

International Standard Randomized Controlled Trial Number (ISRCTN): 58006579; https://www.isrctn.com/ISRCTN58006579

**International Registered Report Identifier (IRRID):**

DERR1-10.2196/22533

## Introduction

### Background

Barth syndrome is a very rare, life-threatening, X-linked recessive genetic disease that almost exclusively affects young males. The causative gene *TAZ* encodes the protein tafazzin, whose aberrant function perturbs the metabolism of the phospholipid cardiolipin (CL). CL is a major constituent of inner mitochondrial membranes and, therefore, majorly affects the muscular tissues that are most reliant on energy production. This results in infantile cardiomyopathy (including stillbirth) and lifelong severe exercise intolerance, lethargy, and fatigue [1]. Low neutrophil numbers (neutropenia), poor feeding, and growth delay are less intuitive but common features [1]. Neutropenia predisposes to serious bacterial infection and is symptomatically treated in two-thirds of UK patients with Barth syndrome via chronic subcutaneous injection therapy with granulocyte-colony stimulating factor—a distressing and expensive medication [2]. Lethargy and fatigue interfere with schoolwork, play, and working life and often necessitate the use of wheelchairs. Tafazzin defects also result in excessive conversion of the mature form of cardiolipin (L4-cardiolipin) to monolysocardiolipin (MLCL), which results in a grossly perturbed cardiolipin ratio that is diagnostic for the disease.

There are no specific treatments for Barth syndrome other than supportive symptomatic care. Overall, 30% of UK patients with Barth syndrome have undergone cardiac transplantation, several of whom have died of related complications. Sudden cardiac death in patients can also occur [3], the possibility of which remains a cause of chronic anxiety in the families of affected persons. Disease-specific therapy is required to prevent morbidity, mortality, psychological distress, disruption of quality of life (QoL), and ameliorate the high socioeconomic burden. Experiments using lymphoblasts from patients with Barth syndrome showed that treatment with either bezafibrate or resveratrol can partially normalize the deranged ratio [4], suggesting their potential as specific therapies.

Resveratrol, a naturally occurring food supplement available from nutraceutical companies, affects energy metabolism and mitochondrial function and has a short half-life in blood. This may explain the lack of consistent clinical efficacy in a range of mammalian and human conditions [5]. In contrast, bezafibrate is well established as a lipid-lowering agent in adults and children with a good safety record for long-term use [6]. Encouragingly, it has been shown to improve left ventricular (LV) function at supraphysiological doses in a Barth knockdown mouse model [7], to protect LV function at a clinically relevant dose [8] and to ameliorate impaired exercise capacity [8]. Therefore, gold standard evidence from a randomized controlled trial (RCT) is now required to investigate the potential risks and benefits of bezafibrate in the population with Barth syndrome. The United Kingdom’s National Health Service (NHS) Specialized Services Barth Syndrome Service (BSS) at University Hospitals Bristol and Weston NHS Foundation Trust (UHBW) is uniquely well positioned to explore such a therapy in an RCT, as it cares for the world’s highest density of diagnosed patients and is the only national multidisciplinary service. The BSS currently cares for 26 living boys and 1 girl (from approximately 200 diagnosed worldwide).

### Objectives

The objective of this study is to determine whether bezafibrate (and/or resveratrol in vitro) will increase mitochondrial biogenesis and potentially modify the cellular ratio of MLCL to L4-CL, ameliorating the disease phenotype in those living with the disease and establishing whether the drug is free of any significant side effects at clinically effective doses. The specific objectives are as follows:

1. Measure the effects of bezafibrate treatment on biochemical and clinical outcome measures and QoL in comparison with placebo in Barth syndrome
2. Correlate clinical improvements with the in vitro analysis of CL ratio and profile and mitochondrial morphology when exposed to bezafibrate and resveratrol in laboratory culture
3. Determine the most feasible methods and standardized outcome measures to allow for better conduct of future trials and evaluations in Barth syndrome
4. Create a research infrastructure that optimizes recruitment, retention, and communication with families and people with Barth syndrome
5. Evaluate participant and family perceptions of research and any important potential barriers to participation.

## Methods

### Trial Design

**Figure 1:**
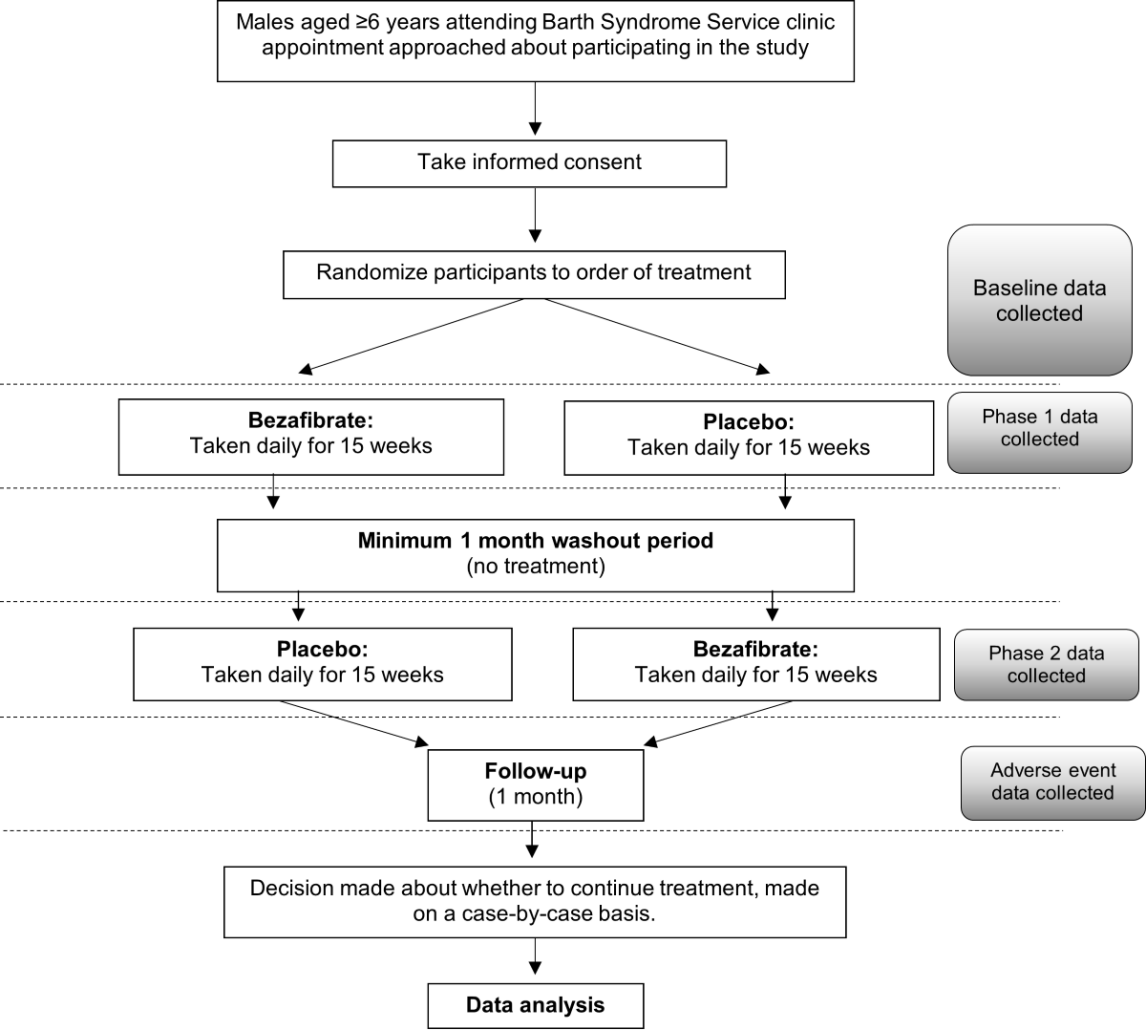
Study schema.

This is a phase 2, UK, single-center, double-blinded, randomized, placebo-controlled crossover study that was conducted at UHBW, a tertiary care research and teaching hospital. Treatment was administered in two 15-week phases with a minimum washout period of 1 month between the phases where no treatment was administered. Participants were followed up for 1 month after the end of the second treatment period (Figure 1).

### Study Population

Males aged ≥6 years in the United Kingdom with a confirmed diagnosis of Barth syndrome were included in this study. Males aged <6 years were not included in this patient population because of the difficulties in obtaining data on the primary outcome through bicycle ergometry in young children.

Patients could enter the study if all the following criteria applied: (1) males aged ≥6 years, (2) clinical diagnosis of Barth syndrome with a characteristic abnormality of the L4-CL/MLCL ratio plus identified mutation in the Tafazzin gene, (3) receiving care from the NHS BSS, (4) stable cardiac condition, and (5) able to swallow bezafibrate tablets (similar size to ibuprofen tablets).

Patients were not able to enter the study if any of the following applied:

1. Known hypersensitivity to bezafibrate, to any component of the product, or to other fibrates
2. Known photoallergic or phototoxic reactions to fibrates
3. Hepatic dysfunction and/or liver function tests greater than 2 times the upper limit of normal.
4. A LV shortening fraction of <25% (or a significant drop in the shortening fraction in the previous year)
5. Documented atrial or ventricular arrhythmia (atrial/ventricular tachycardia or atrial/ventricular fibrillation) that had not been stabilized with treatment
6. Renal impairment (defined as creatinine clearance <90 mL/min)
7. Preexisting known gallbladder disease
8. Recent unspecified significant deterioration in general health
9. Prisoners and adults lacking capacity to provide informed consent.

There have been reports of rhabdomyolysis occurring in patients treated with a combination of bezafibrate and statins. Therefore, with agreement from the patients’ cardiologists, patients who underwent cardiac transplantation and were administered statins ceased their statin medication 2 weeks before their trial participation.

### Randomization

Using blocks of undisclosed size, random sequence allocations of bezafibrate or placebo were generated by a computer before the start of the study by an independent statistician. The allocation sequence, concealed from all clinical and research personnel, was then attached to a list of consecutive study IDs and provided directly to the UHBW Pharmacy Trials Unit (PTU), which dispensed the study medication. The allocations of the drug or placebo in the second phase of the trial were opposite to those in the first phase. Once consent had been granted for a participant and eligibility had been established and confirmed, they were allocated to the next consecutive study ID and the medication prescribed and dispensed according to the study ID and trial phase.

### Trial Interventions

Many of the potential participants had difficulty in swallowing large tablets; therefore, to recruit and retain as many participants as possible, we commissioned the manufacturing of small 100 mg bezafibrate and placebo tablets for the trial. All participants received 15 weeks of the intervention (bezafibrate) and 15 weeks of placebo. The order in which bezafibrate and placebo were administered depended on the first allocated treatment at randomization. Both the bezafibrate and placebo arms had a minimum washout period (without treatment) of 1 calendar month before starting the alternative treatment arm. The study intervention was prescribed once at the start of the study and again at the end of phase 1 (for the forthcoming phase 2 period). Participants administered their own medication at home, according to the prescribed regimen detailed below. The trial prescription did not specify the medication to be taken, as this was determined by the randomization list provided to UHBW PTU.

Bezafibrate was taken orally in a tablet formulation (100 mg tablets, standard release formulation):

- Children aged 6-9 years: commenced on 100 mg omne in die (ie, once a day, OD) for the first month, and if well tolerated, increased to 100 mg BD (ie, twice a day, bis in die) for the remaining 3-month treatment period.
- Children aged 10-17 years: commenced on 200 mg OD for the first month, and if well tolerated, increased to 200 mg BD for the remaining 3-month treatment period.
- Adults (≥18 y): 200 mg BD.

The placebo was a visually identical tablet containing a formulation with no active substance, taken orally as per the number of tablets and frequency described above.

Participants remained on the initial loading dose until they were instructed to increase the dose after a satisfactory assessment of safety by the principal investigator. Serious adverse events (SAEs) could prompt an immediate withdrawal of the drug for a period at the discretion of the treating physician. Guidance on the use (and any restrictions) of concomitant medications is available in the full protocol. Once participants completed the study treatment, their medical care was reverted to the standard care received from the BSS.

Adherence to study treatment was assessed by patient-reported missed doses and returned unused medications. During monthly follow-up telephone calls, the participants (or the carers of young children) were asked if any doses were missed and, if so, the number of missed doses was recorded. Participants were also asked to return all their study medication bottles at the end of each treatment phase. Unused tablets were counted and compared with the expected number of tablets to be taken. All tablets were assumed to be taken if the bottle was empty. Participants were classified as adherent if they took at least 70% of their tablets.

### Statistical Analysis Plan

All participants completed and contributed data to both phases of the crossover study, irrespective of any periods when the intervention was suspended because of ill health. The primary analyses will adhere to the intention-to-treat principle, but we envisage that additional secondary or sensitivity analyses may be specified in a detailed analysis plan, written before completing the final data queries for final analyses. The data will be analyzed to follow the reporting guidelines of Consolidated Standards of Reporting Trials.

Our plan for analyses assumes that the continuously scaled outcome data (or transformed data) will be distributed satisfactorily to allow parametric methods to be applied. We aim to analyze the data using mixed-effects regression methods that will allow participants’ data to be included if not complete. The regression models will estimate the effects for both the treatment factor (2 levels) and period of intervention (2 levels), adjusting for the baseline assessments of outcomes at the time of recruitment. Participants will be fitted as random effects. We will test for the presence of a carryover effect (ie, the possibility that results obtained during the second treatment phase are affected by what happened in the first treatment phase) by including a treatment-by-time period interaction in the model. We do not expect any statistical evidence of an interaction.

Similar methods will be used for analyses of the additional objectives to ensure that (1) the data hierarchy is respected (ie, repeated measurements within subjects) and (2) available data for all participants can be included. Regression models will be fitted to estimate the differences in secondary outcomes when treated with bezafibrate versus placebo and, additionally, to quantify the extent to which more *proximal* biomarkers are associated with more *distal* clinical and symptomatic outcomes. Nonadherence to random allocations will be documented, and every effort will be made to include all the randomized participants.

Access to study data will be limited to authorized personnel. The data will be collected and retained in accordance with the UK General Data Protection Regulation 2018. An anonymized data set will be held for future research, as per the National Institute for Health Research (NIHR) contractual arrangements.

### Primary and Secondary Outcomes

The primary outcome measure is the change in peak volume oxygen consumption (peak VO_2_) on bicycle ergometry from baseline to the final week of each treatment phase, as this is strongly associated with activity intolerance and may correlate with subjective fatigability, which is the most important determinant of QoL.

Secondary outcomes are (1) MLCL/L4-CL ratio/CL profile in blood cells, (2) PCr/ATP ratio in cardiac muscle on phosphorus-31 (31P) magnetic resonance spectroscopy (MRS), (3) skeletal muscle oxidative function on 31P MRS, (4) QoL assessed using age-appropriate PedsQL (Pediatric Quality of Life Inventory) questionnaires, (5) absolute neutrophil count, (6) amino acid expression (serum arginine and cysteine levels), (7) cardiac function (including left ventricular ejection fraction [LVEF] and 2D LV mean peak systolic strain), (8) mitochondrial size in lymphocytes, (9) number of mitochondria (per lymphocyte), (10) total area of mitochondria per lymphocyte, (11) area of mitochondria as the proportion of cytoplasm, (12) mitochondria function and cristae organization in lymphocytes or neutrophils, and (13) arrhythmia profile from 12-lead electrocardiogram (ECG) at rest and during exercise (for potential rhythm abnormalities).

In addition, we will integrate qualitative research methods to explore participants’ and families’ experiences of the different interventions and their participation perceptions. Parents of younger participants (<18 y) and participants (>14 y) will be invited to participate in semistructured one-to-one interviews during the assessment periods after the first and second interventions.

The validity of the outcome measurements is guaranteed by placebo control and blinding, providing this is not broken. In addition, all measurements were obtained according to the standard hospital or outcome-specific protocols. Stress echocardiography and VO_2_ measurements were obtained using a modified McMaster protocol (Multimedia Appendix 1 [9]). Cardiolipin measurements were obtained using a matrix-assisted laser desorption/ionization mass spectroscopy protocol (Multimedia Appendix 2), and the characterization and control of mitochondrial morphology were performed as previously described by Acehan et al [10], using electron microscopic tomography. Interobserver variability was also minimized, where possible, by obtaining data using the same study personnel. For example, electron microscopy data were obtained by a single laboratory technician.

### Data Collection

The data collection schedule for this study is shown in Table 1. Data were collected on paper *Case Report Forms* and then entered into a password-protected and access-restricted electronic spreadsheet. A second spreadsheet was created for independent double data entry to minimize data entry errors and opportunities for manipulation.

Throughout the study, monthly blood tests were taken at the patients’ local hospital or general practitioner’s surgery to assess the ongoing safety of treating participants with bezafibrate.

**Table 1:** Data collection for trial participants.

Event		Baseline	Phase 1					Washout period		Phase 2					Follow-up
		Month 0	Month 1	Month 2	Month 3	Month 4	Month 5		Month 6		Month 7	Month 8	Month 9	Month 10	
Informed consent		✓^a^	N/A^b^	N/A	N/A	N/A	N/A		N/A		N/A	N/A	N/A	N/A	
Height and weight		✓	N/A	N/A	N/A	✓	N/A		N/A		N/A	N/A	✓	N/A	
Medical history		✓	N/A	N/A	N/A	N/A	N/A		N/A		N/A	N/A	N/A	N/A	
Clinical examination^c^		✓	N/A	N/A	N/A	✓	N/A		N/A		N/A	N/A	✓	N/A	
**Bicycle ergometry (exercise bike test)**															
	Peak oxygen consumption	✓	N/A	N/A	N/A	✓	N/A		N/A		N/A	N/A	✓	N/A	
	Tissue Doppler studies	✓	N/A	N/A	N/A	✓	N/A		N/A		N/A	N/A	✓	N/A	
Echocardiogram (at rest and during exercise)^d^		✓	N/A	N/A	N/A	✓	N/A		N/A		N/A	N/A	✓	N/A	
12-lead ECG^e^ at rest and during exercise (during echo)		✓	N/A	N/A	N/A	✓	N/A		N/A		N/A	N/A	✓	N/A	
**Blood sample (20-30 mL total)^f^**															
	Transformed lymphoblast line for in vitro incubation with bezafibrate and resveratrol	✓	N/A	N/A	N/A	N/A	N/A		N/A		N/A	N/A	N/A	N/A	
	FBC^g^, absolute neutrophil count, urea/electrolytes, LFT^h^, CK^i^, plasma arginine/cysteine, full lipid profile (total cholesterol, high-density lipoprotein, and triglycerides), and brain natriuretic peptide	✓	N/A	N/A	N/A	✓	N/A		N/A		N/A	N/A	✓	N/A	
	Mitochondrial assessment	✓	N/A	N/A	N/A	✓	N/A		N/A		N/A	N/A	✓	N/A	
**Blood sample (safety assessment) done locally to the patient**															
	FBC including absolute neutrophil count, routine renal and LFT, plasma triglyceride/total cholesterol/LDL^j^-cholesterol and creatine kinase, and creatinine	N/A	✓	✓	✓	N/A	✓		✓		✓	✓	N/A	N/A	
Cardiac/skeletal muscle MRI^k,l^/MRS^m,n^ scan		✓	N/A	N/A	N/A	✓	N/A		N/A		N/A	N/A	✓	N/A	
PedsQL^o^		✓	N/A	N/A	N/A	✓	N/A		N/A		N/A	N/A	✓	N/A	
Adverse events		N/A	✓	✓	✓	✓	✓		✓		✓	✓	✓	✓	
Drug prescribing		✓	N/A	N/A	N/A	✓	N/A		N/A		N/A	N/A	N/A	N/A	
Qualitative interview		N/A	N/A	N/A	N/A	✓	N/A		N/A		N/A	N/A	✓	N/A	

^a^Data was collected (or event took place) at this time.

^b^N/A: not applicable.

^c^Including resting blood pressure, heart rate, and oxygen saturation.

^d^Cardiac status: left ventricular ejection fraction and 2D strain.

^e^ECG: electrocardiogram.

^f^Samples for detailed cardiolipin profiling and calculation of MLCL/L4-CL ratio; examination of mitochondria of the blood cells by electron microscopy; measurements of size, number, and shape recorded; and the analysis of mitochondrial function studies including the analysis of respiratory chain enzyme complexes.

^g^FBC: full blood count.

^h^LFT: liver function test.

^i^CK: creatine kinase.

^j^LDL: low-density lipoprotein

^k^MRI: magnetic resonance imaging.

^l^Cardiac magnetic resonance imaging will be used to assess right and left ventricular functions and volumetrics.

^m^MRS: magnetic resonance spectroscopy.

^n^Magnetic resonance spectroscopy will be used to assess phosphocreatine to adenosine triphosphate ratio and oxidative function (phosphocreatine recovery kinetics) in cardiac and skeletal muscles.

^o^PedsQL: Pediatric Quality of Life Inventory.

### Blinding and Code Breaking

Neither the participants nor investigators (or any other member of the research team apart from the pharmacist) knew about the allocated treatment being administered or the order in which it was administered. The placebo was visually identical to the bezafibrate tablets and was as similar as possible in taste and smell. The tablets containing bezafibrate or placebo did not have a strong or unusual smell or taste, which minimized the unblinding of participants and subsequently the investigators. Therefore, we did not anticipate any unblinding because of the characteristics of the drug. However, bezafibrate may have induced side effects in some participants that could have inadvertently unblinded them, and we acknowledge that this may be a limitation of the study.

Members of the participants’ health care team could request unblinding of the study medication in either phase in the event of a participant experiencing a SAE, if they considered that the information would alter their management of the SAE. Participants were given an unblinding card to carry with them containing instructions for the attending doctor on how to request unblinding (24 hours). All instances of unblinding requests were documented, including who requested the unblinding, why it was required, the time and date, and who performed the unblinding.

### Sample Size Calculation

The number of study participants are clearly limited because of the rarity of the disease. A total of 20 males aged between 6 and 24 years currently attend the NHS National BSS at University Hospitals Bristol and Weston NHS Trust. We anticipated that between 12 and 15 UK patients would be eligible and consent to take part in the trial.

In a simple crossover trial (a single intervention and control comparison), the SD of the within-subject difference between treatments is given as follows: square root (2×within-subject SD) [11]. In addition, all participants constitute their own control. In this trial, the difference in the mean peak oxygen consumption between the placebo and bezafibrate phases will be tested, assuming a 2-tailed alpha of .05. For a sample size of 12 participants, the trial will be able to detect a difference of 0.90 (within subject) SD with 80% power or 1.05 SD with 90% power.

### Research Procedures

Eligibility was assessed and written informed consent was obtained by a medically qualified doctor at the baseline research clinic visit in Bristol. Patients who did not live near Bristol were offered accommodation so that they (and their families) could attend the research clinics without incurring additional expenses. Detailed assessments were performed at baseline clinic visits and at the end of each treatment phase. The assessments were timed during the final week of therapy so that participants were still receiving the intervention or placebo at the time of testing but had maximum cumulative exposure to the drug or placebo. Assessments included the following:

- Anthropometric data (height and weight)
- Medical history and examination (including resting blood pressure, heart rate, and oxygen saturation)
- Transthoracic echocardiographic determination of cardiac function at rest using ejection fraction, pulsed wave tissue Doppler, and 2D myocardial strain at rest and during quantitative exercise stress echo [12]
- Modified McMaster exercise protocol to assess the peak oxygen consumption using an electronically braked General Electric health care exercise echocardiography couch
- PCr/ATP ratio and oxidative function (PCr recovery kinetics) in cardiac and skeletal muscle using MRS
- QoL questionnaires (PedsQL) [13]
- Qualitative assessments (semistructured one-to-one interviews) to assess patients’ experiences of the intervention and participating in a trial
- Cardiac arrhythmia profile from 12-lead ECG at rest and during exercise
- Cardiac magnetic resonance imaging (Siemens 3T Magnetom Skyra) to assess the ventricular function and volumetrics (regional wall motion abnormalities).

In total, 20 mL of blood was taken at each assessment visit for the following tests:

1. Full blood count, absolute neutrophil count, urea and electrolytes, liver function tests, creatine kinase, plasma arginine and cysteine, full lipid profile (total cholesterol, high-density lipoprotein, and triglycerides), and brain natriuretic peptide (as a blood marker of LV function)
2. Mitochondrial tests: assessment of detailed CL profiling and calculation of the MLCL/L4-CL ratio. The mitochondria of the blood cells were examined by electron microscopy for lymphocytes or neutrophils, and the measurements of size, number, and shape were recorded. Neutrophils or lymphocytes extracted from 5-mL whole blood were analyzed for mitochondrial function, including the analysis of respiratory chain enzyme complexes using established spectrophotometric methods.

At baseline, the establishment of an Epstein-Barr virus (EBV)–transformed lymphoblast line for in vitro incubation with resveratrol or bezafibrate and the assessment of CL ratio also occurred. Some participants had existing EBV cell lines available from a separate study. Rather than trying to establish new cell lines from these participants (a time-consuming and not always successful process), we sought consent to use their existing cell lines for this study.

Subjective QoL will be assessed using age-appropriate PedsQL assessment forms (core and fatigue scales) and parental questionnaires. These include forms suitable for young adults (18-25 y).

### Patient and Public Involvement

The study was conceptualized with inputs from patient and family stakeholders, including the chair of the UK Barth Syndrome Trust (BST), who is a coapplicant of the study. She advised on the acceptability of the study design for potential participants, helped arrange clinic dates with prospective families, and optimized the groups of individuals in each clinic to increase the likelihood of engagement and retention. In addition, a member of the public with experience of Barth syndrome is a member of the Trial Steering Committee (TSC), advising on aspects where family views are needed.

Both the TSC member and the Chairperson of the BST will be involved in reviewing the information about study results that will be disseminated to the Barth syndrome community. Study findings will be disseminated to the Barth syndrome community through a joint press release by the research team and the patient or family stakeholders (BST) and on a patient and family study day. The final study report will be published in the open-access NIHR Journals Library.

### Regulatory Approvals

Research ethics approval was granted by the UK (South West–Central Bristol) National Research Ethics Service Committee (reference 15/SW/0228) on November 12, 2015. Bezafibrate also comes under the regulation of the Medicines and Healthcare products Regulatory Agency (MHRA), as it is classified as an Investigational Medicinal Product. MHRA approval was obtained on November 3, 2015 (Eudract Number: 2015-001382-10).

### Trial Management

The trial is managed by the Bristol Trials Centre (Clinical Trials and Evaluation Unit) and sponsored by the UHBW. Participants had the right to withdraw at any time. Data collected until the time of withdrawal were included in the analyses, unless the participant expressed a wish for their data to be destroyed. Participants who withdrew from the study continued to be treated according to the standard procedures (ie, management of symptoms only).

### Changes to the Protocol Since It Was First Approved

The exclusion criteria were updated with the following additional items before commencement of recruitment: (1) known hypersensitivity to bezafibrate, to any component of the product, or to other fibrates and (2) known photoallergic or phototoxic reactions to fibrates.

On request of the funding body, an interim analysis was planned during the washout phase of the trial. This was not supported by the TSC or the Data Monitoring and Safety Committee and was subsequently removed.

Other amendments to the protocol were (1) the addition of participant unblinding cards, (2) the inclusion of neutrophil cells on which to perform mitochondrial function tests, (3) the removal of collection of near-infrared spectroscopy data (these were found to be too unstable during the exercise tests), (4) change to the treatment period from 4 calendar months to 15 weeks, (5) clarification of the estimated glomerular filtration rate formula to be used for pediatric participants, and (6) change to the cardiac function measurement from shortening fraction to 2D strain. Several other minor clarifications and administrative updates were made to the protocol. Version 5.0 (dated October 15, 2019) of the protocol is currently in use. Relevant regulatory approvals were obtained for all the amendments to the protocol.

## Results

Twelve patients were scheduled to participate across three consecutive research clinics between March and April 2019. In total, 11 participants were recruited, and follow-up was completed in January 2020. Data analysis is ongoing, with publication expected in early 2021. The full protocol is available [14].

## Discussion

Funding for this study was awarded by the NIHR in relation to a commissioned call for research on interventions for very rare diseases (defined as a disease that affects less than 1 in 100,000 people). This followed a consultation by the United Kingdom’s Department of Health, which found that people with rare diseases face multiple barriers to receiving appropriate care. Most barriers are a consequence of limited scientific and clinical knowledge of the disease, which leads to delays in diagnosis and few treatments of proven effectiveness. The commissioned call demonstrates a drive to improve health care for people with very rare diseases in the United Kingdom. It also highlights the historic dearth of experience in conducting randomized trials in this area. The feasibility of the CARDIOMAN (Cardiolipin Manipulation) study will help to inform the future conduct of RCTs of treatments for populations with rare diseases as well as testing the efficacy of bezafibrate as a potential treatment for the disease and advancing the mechanistic understanding of Barth syndrome.

## Appendix

Multimedia Appendix 1 Stress echocardiography and modified McMaster protocol.

Multimedia Appendix 2 Matrix-assisted laser desorption/ionization mass spectroscopy protocol for cardiolipin outcomes.

Multimedia Appendix 3 SPIRIT checklist.

## Abbreviations

31P: phosphorus-31
ATP: adenosine triphosphate
BSS: Barth Syndrome Service
BST: Barth Syndrome Trust
EBV: Epstein-Barr virus
ECG: electrocardiogram
L4-CL: L4-cardiolipin
MHRA: Medicines and Healthcare products Regulatory Agency
MLCL: monolysocardiolipin
MRS: magnetic resonance spectroscopy
NHS: National Health Service
NIHR: National Institute for Health Research
OD: omne in die
PCr: phosphocreatine
PedsQL: Pediatric Quality of Life Inventory
PTU: Pharmacy Trials Unit
QoL: quality of life
RCT: randomized controlled trial
SAE: serious adverse event
TSC: Trial Steering Committee
UHBW: University Hospitals Bristol and Weston NHS Foundation Trust
VO_2_: volume oxygen (consumption)
