# Supplementary material for: Treatment of Barth Syndrome by Cardiolipin Manipulation (CARDIOMAN) With Bezafibrate: Protocol for a Randomized Placebo-Controlled Pilot Trial Conducted in the Nationally Commissioned Barth Syndrome Service
Source: JMIR Res Protoc. 2021 May 31;10(5):e22533. doi: 10.2196/22533 (PMC8204243; doi:10.2196/22533)
Supplement: Multimedia Appendix 1 [file resprot_v10i5e22533_app1.pdf]

Appendix 1

Stress echocardiography and modified McMaster protocol.

Participant's baseline heart rate (HR) and blood pressure (BP) are recorded at rest and at peak exercise (Dinamap Pro 100V2, GE Medical Systems Information Technologies 2002, Tampa, Florida, USA).

Exercise stress test is performed on an electronically braked GE Healthcare exercise echocardiography eBike EL (GE Medical Systems Information Technologies GmbH, Freiburg, Germany) whilst echocardiography is simultaneously performed by a cardiologist. A step protocol is used, with each child beginning on a baseline of 0 watts, and the intensity increased by 25 watts every 3 minutes (modified McMaster protocol). Exercise stages of three minutes are used to obtain detailed "steady state" information for each exercise stage and facilitate echocardiographic image acquisition. Children are encouraged to cycle at 60 revolutions per minute (rpm) throughout. Children are told to cycle until completion/ the point of voluntary exhaustion. The exercise test is terminated when the child is unable to continue due to exhaustion or when they cannot sustain a cadence of 60 ± 5 rpm.

Oxygen consumption is measured using a gas cart (Metalyzer 3B Cortex, Biophysik, Leipzig, Germany). The device was calibrated using a known reference gas before testing and a 3 litre calibration syringe was used to calibrate the turbine volume transducer (Hans Rudolph, Kansas City, MO). The gas cart provided breath-by-breath gas exchange analysis, averaged to 10 second time intervals. The highest 10 second average VO_2 represented that individuals VO_2peak. Average data for each stage is expressed as the average of the last 30 seconds of each workload. The Borg Scale is used to measure the rating of perceived exertion (RPE) at the end of each 3 minute interval, with the children asked to subjectively indicate which grading (scale out of 10) is relevant to them at that point in the test.

Functional trans-thoracic echocardiography at rest is performed by a cardiologist following the guideline of the American Society of Echocardiography [1] and using the following hardware: Vivid Q or E9, General Electric, cardiac transducers 4-6 Mhz, GE Healthcare, Little Chalfont, UK). Left Ventricular (LV) systolic function during rest and exercise is assessed using short axis and long axis fractional shortening (FS), Pulse Wave –Tissue Doppler Imaging (PW-TDI) derived myocardial systolic velocities and 2-D myocardial strain analysis (speckle tracking). For longitudinal and radial strain analysis images of the left ventricle are obtained in apical 4 chamber and parasternal short axis views. Images for strain analysis are obtained at a rate of 40-90 frames per minute during rest and exercise. Diastolic LV function is assessed using Pulse Wave –Tissue Doppler Imaging (PW-TDI) derived myocardial diastolic velocities and PW Doppler mitral valve inflow velocities. Fast image acquisition during exercise is facilitated using the GE exercise stress protocol GE software. Functional echocardiographic quantification of left ventricular function is processed offline using the Q analysis research tool of ECHO PAC offline software (EchoPac, GE Healthcare, Little Chalfont, UK).

Echocardiography protocol:

Rest:

Exercise and recovery: 7 images per exercise stage:

1. 2-D 4 Chamber view LV

2. Tissue Doppler Imaging (TDI) 4 Chamber view

3. Pulse Wave (PW) TDI lateral LV 4 chamber view
4. Mitral valve inflow PW Doppler trace
5. Aortic outflow PW Doppler trace
6. 2-D parasternal short-axis view (base)
7. 2-D parasternal short axis view (apex)

References:

1. Lopez L, Colan SD, Frommelt PC, Ensing GJ, Kendall K, Younoszai AK, et al. Recommendations for quantification methods during the performance of a pediatric echocardiogram: a report from the Pediatric Measurements Writing Group of the American Society of Echocardiography Pediatric and Congenital Heart Disease Council. J Am Soc Echocardiogr. 2010 May;23(5):465-95; quiz 576-7.
