# Supplementary material for: Treatment of Barth Syndrome by Cardiolipin Manipulation (CARDIOMAN) With Bezafibrate: Protocol for a Randomized Placebo-Controlled Pilot Trial Conducted in the Nationally Commissioned Barth Syndrome Service
Source: JMIR Res Protoc. 2021 May 31;10(5):e22533. doi: 10.2196/22533 (PMC8204243; doi:10.2196/22533)
Supplement: Multimedia Appendix 2 [file resprot_v10i5e22533_app2.pdf]

**Appendix 2**

**MALDI Mass Spectroscopy protocol for cardiolipin outcomes**

Total protein concentrations for all samples were measured using the Pierce bicinchoninic acid (BCA) protein assay kit following the manufacturer's instructions. All protein assays were carried out in triplicate. An aliquot with an equivalent volume for 20 mg of protein was extracted. The sample was centrifuged at 16,000 g for 30 seconds, the supernatant discarded and 10 ml of $CHCl_3$ added to the pellet and vortexed to extract the lipids. The matrix solution of 9-aminoacridine (9-AA) in 2-propanol/acetonitrile (60/40, v/v) at a concentration of 10 mg/ml was used in all cases. A 10 ml aliquot of the matrix solution was added to the $CHCl_3$ lipid extracted pellet and the sample mixed. A 0.4 mM solution of tetra-myristoyl cardiolipin CL $(14:0)_4$ (Avanti polar lipids) in $CHCl_3$ was prepared and used as an internal standard. A 0.5 ml aliquot of the internal standard solution was added to the lipid extract-matrix mixture and this solution centrifuged at 16,000 g for 30 seconds. The lipid extract matrix solution was then spotted onto the polished steel MALDI target in droplets of 0.35 ml and analysed. MALDI-TOF mass spectra of intact lipids were acquired on Bruker Ultraflex mass spectrometer (Bruker Daltonics, Bremen, Germany). Instrument performance and calibration was routinely checked against a known mixture of peptides in the mass range 900 to 3600Da (AB Sciex Calmix 2). The calibrant solution was prepared as per the manufacturer recommendations using CHCA as a matrix in acetonitrile/water (7/3, v/v) with 0.1% trifluoroacetic acid as an additive.
